# Extraskeletal myxoid chondrosarcoma: tumor response to sunitinib

**DOI:** 10.1186/2045-3329-2-22

**Published:** 2012-10-11

**Authors:** Silvia Stacchiotti, Gian Paolo Dagrada, Carlo Morosi, Tiziana Negri, Antonella Romanini, Silvana Pilotti, Alessandro Gronchi, Paolo G Casali

**Affiliations:** 1 Department of Cancer Medicine, Adult Sarcoma Medical Oncology Unit, Fondazione IRCCS Istituto Nazionale Tumori Milan, via Venezian 1, 20133, Milan, Italy; 2 Department of Pathology and Laboratory of Molecular Pathology, Fondazione IRCCS Istituto Nazionale Tumori Milan, Milan, Italy; 3 Department of Radiology, Fondazione IRCCS Istituto Nazionale Tumori Milan, Milan, Italy; 4 Oncology Department, University Hospital Santa Chiara, Pisa, Italy; 5 Department of Surgery, Fondazione IRCCS Istituto Nazionale Tumori Milan, Milan, Italy

**Keywords:** Sarcoma, Myxoid extraskeletal chondrosarcoma, Sunitinib malate, Targeted therapy, Chemotherapy

## Abstract

**Background:**

Extraskeletal myxoid chondrosarcoma (EMCS) is a rare soft tissue sarcoma of uncertain differentiation, characterized in most cases by a translocation that results in the fusion protein *EWSR1*-*CHN* (the latter even called *NR4A3* or *TEC*). EMCS is marked by >40% incidence of metastases in spite of its indolent behaviour. It is generally resistant to conventional chemotherapy, and, to the best of our knowledge, no data have been reported to date about the activity of tirosin-kinase inhibitor (TKI) in this tumor. We report on two consecutive patients carrying an advanced EMCS treated with sunitinib.

**Methods:**

Since July 2011, 2 patients with progressive pretreated metastatic EMCS (Patient1: woman, 58 years, PS1; Patient2: man, 63 years, PS1) have been treated with continuous SM 37.5 mg/day, on an individual use basis. Both patients are evaluable for response. In both cases diagnosis was confirmed by the presence of the typical *EWSR1-CHN* translocation.

**Results:**

Both patients are still on treatment (11 and 8 months). Patient 1 got a RECIST response after 4 months from starting sunitinib, together with a complete response by PET. An interval progression was observed after stopping sunitinib for toxicity (abscess around previous femoral fixation), but response was restored after restarting sunitinib. Patient 2 had an initial tumor disease stabilization detected by CT scan at 3 months. Sunitinib was increased to 50 mg/day, with evidence of a dimensional response 3 months later.

**Conclusions:**

Sunitinib showed antitumor activity in 2 patients with advanced EMCS. Further studies are needed to confirm these preliminary results.

## Introduction

Extraskeletal myxoid chondrosarcoma (EMCS) is a rare soft-tissue sarcoma (STS) first described in 1972 [1,2]. EMCS is now considered a unique entity of uncertain differentiation (convincing evidence of a cartilaginous differentiation is lacking in most cases) [3]. Besides, EMCS can have a neuroendocrine differentiation [4-6].

It is market by a specific chromosomal translocation, t(9;22)(q22.3;q12.2), fusing *CHN* to *EWSR1*[7,8]. Less frequently two different translocations, t(9;17)(q22.3;q12) and t(9;15)(q22.3;q21.3) are found, resulting in *RBP56CHN* and *TCF12CHN* fusion-genes, respectively [9,10]. The fusion-proteins promote cellular growth and differentiation [11]. Furthermore, the EWSR1-*CHN* fusion-protein may activate the *PPARG* nuclear receptor gene [12]. Microscopically, EMCS can be subdivided into a conventional well-differentiated and a cellular high-grade EMCS, which is marked by epithelioid cells with prominent nucleoli, high mitotic rate and necrosis [13]. Dedifferentiated ECMS were also described [14].

Most EMCS arise from the deep soft tissues of the extremities and limb girdles [15-20]. The natural history is usually characterized by an indolent behavior, but studies with a long median follow-up show a high-rate of late local and distant tumor recurrence despite a prolonged clinical course [16-21]. Metastases are reported in around 40% of cases, with a 58% overall survival rate at 15 years in the largest retrospective series [20].

Standard treatment of primary EMCS is complete surgical resection, followed by radiation in high-risk cases [21]. Patients with advanced disease usually receive a medical treatment. Unfortunately, response rates to conventional chemotherapy are low [15,21-24].

### Case report

**Figure 1:**
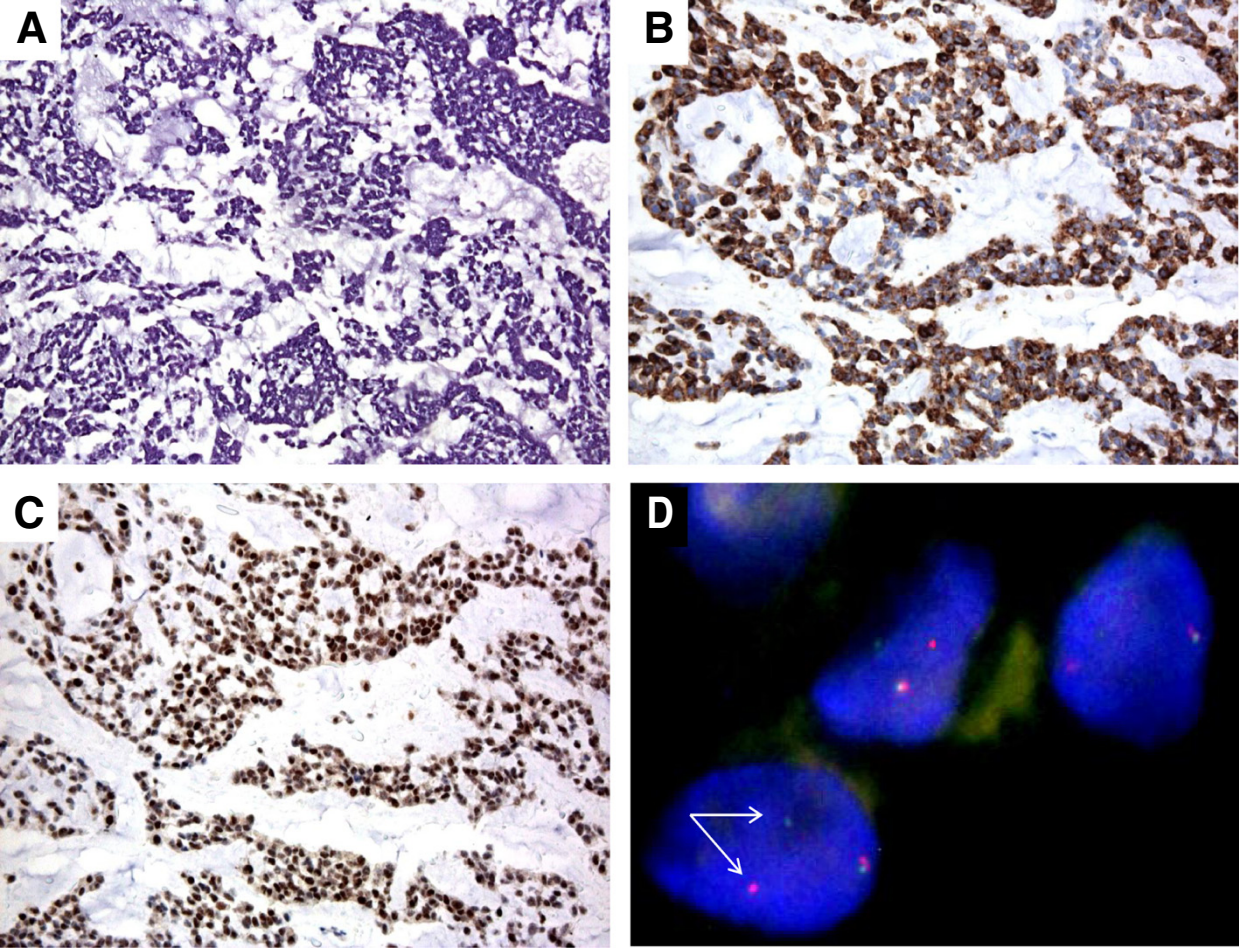
**Patient 1 tumor biopsy consistent with the diagnosis of extraskeletal myxoid chondrosarcoma.** Histology shows nests/cords of epithelioid cells embedded into a myxoid matrix (HandE) (panel **A**), characterized by diffuse pancytokeratin (AE1/AE3) cytoplasmic decoration (panel **B**) and PPARG nuclear expression (panel **C**). Panel **D** shows the FISH rearranged pattern for *CHN*, consisting in a fusion green/red signal, corresponding to an intact copy of the gene, and a split signal (red and green separated, white arrows) corresponding to the rearranged allele.

A 53-year female patient was diagnosed with a 20-cm mass located to the right thigh in October 2006. Concomitant ipsilateral iliac lymph nodes (LN) were present. Both sites were biopsied, with a pathologic diagnosis of EMCS (Figure 1). In both samples histology showed a diffuse pancytokeratin (AE1/AE3) cytoplasmic staining and PPARG nuclear expression, whileS-100 protein, smooth muscle actin, synaptophysin and brachyury were negative. *EWSR1* and *CHN* gene status was investigated by dual-color break-apart FISH which detected a balanced rearrangement for both genes. Figure 1D shows the FISH rearranged pattern for *CHN*.

The identification of a *CHN* translocation partner ruled-out the diagnosis of myoepithelial carcinoma, raised by morphology and cytokeratin immunoreactivity, as well as an ossifying fibromixoid tumor, another ECMS mimic [25]. Indeed, a subset of soft tissue myoepithelial carcinomas were recently reported to harbour *EWSR1-POU5F1* chimera [26] widening the growing family of *EWSR1* gene-rearranged tumors and thus making the *EWSR1* rearrangement test alone unable to discriminate.

The patient was treated with anthracycline-based chemotherapy. This was followed by a wide excision of the primary tumor en-bloc with a partial femur resection plus ipsilateral iliac LN dissection, and adjuvant radiotherapy on the tumor bed and on the right iliac region. A first multifocal intrabdominal/retroperitoneal relapse was detected in February 2008, and was treated with high-dose ifosfamide plus complete surgical resection. In December 2009, lung metastases appeared. A third-line chemotherapy with trabectedin was started, with progression. Lacking any conventional alternative, in June 2011 the patient started sunitinib 37.5 mg/day, on a continuous dosing regimen. Treatment was provided within a compassionate use program, with the approval of the Institutional Ethics Committee. At that time the disease involved the lungs, liver, abdomen, soft tissues, as confirmed by computed tomography scan (CT) and [18F]fluorodeoxyglucose–positron emission tomography scan (PET). No evidence of relapse to the primary tumor site was observed. Patient was asymptomatic, ECOG performance status (PS) = 1.

**Figure 2:**
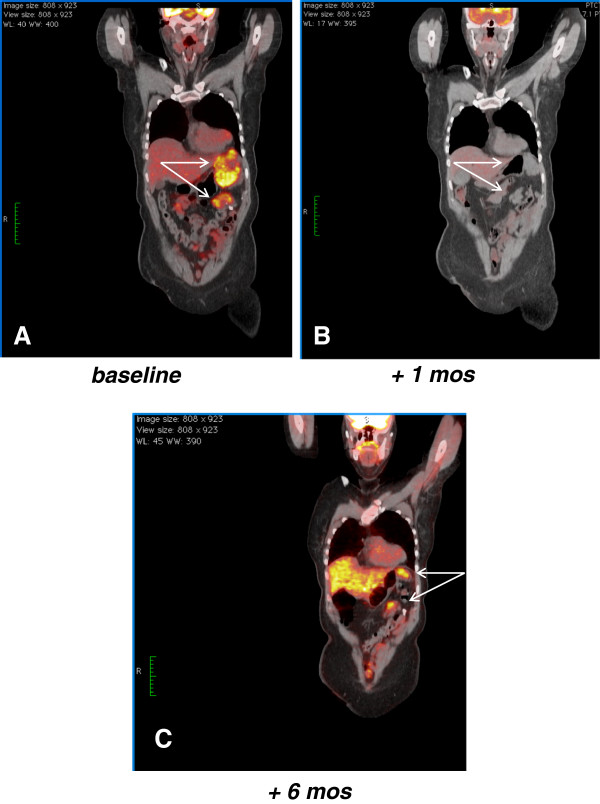
**[18F]fluorodeoxyglucose–positron emission tomography scan (PET) tumor assessment.** In panels **A** and **B** (white arrows) PET shows a marked decrease in maximum SUV comparing the tumor before and after 4 weeks of treatment. Panel **C** shows an interval tumor progression at 6 months from baseline, i.e. after 2 months from sunitinib interruption and treatment with antibiotics.

**Figure 3:**
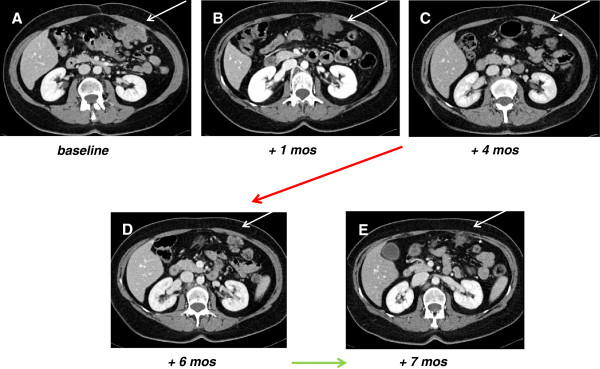
**Computed Tomography scan (CT) tumor assessment.** In panels **A**, **B**, **C** (white arrows) CT, venous phase, after contrast medium, shows a peritoneal metastasis at baseline, after 1 and 4 months of sunitinib with evidence of 30% decrease in tumor size and contrast uptake. Panel **D** shows an interval tumor progression at 6 months from baseline, i.e. after 2 months from sunitinib interruption, while panel **E** shows a new response at 7 months, 4 weeks after restoring sunitinib.

A complete response by PET to all lesions was evident after 4 weeks of treatment (Figure 2B) with a marked decrease in maximum SUV, along with a decrease in tumor size and contrast uptake on CT (Figure 3B).

**Figure 4:**
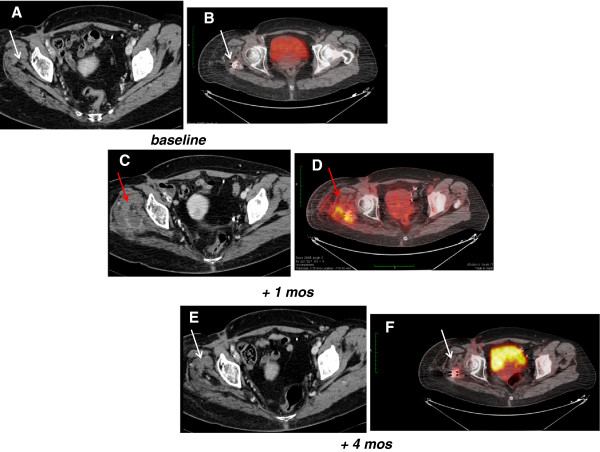
**Computed Tomography scan (CT) and [18F]fluorodeoxyglucose–positron emission tomography scan (PET) abscess evaluation.** CT showed a new mass in the right gluteus muscles characterized by fluid content with rim enhancement (panel **C**) which was not evident at baseline (panel **A**). That area became positive on PET (panel **D**) compared to baseline (panel **B**). CT (panel **E**) and PET (panel **F**) confirmed the resolution of the infection, three months later after sunitinib interruption and treatment with antibiotics.

Unfortunately, 4 weeks after starting treatment, therapy was complicated by an abscess to the primary site, around femoral fixation. The infection was confirmed by clinical signs (fever, leukocytosis, redness, calor and pain located to the thigh), CT and PET. In fact, CT showed a tumor response to all sites, as described above, together with a new mass in the right gluteus muscles characterized by fluid content with rim enhancement (Figure 4C) which was not evident at baseline (Figure 4A). It was located below the painful right tight skin area. Consistently, that area became positive on PET (Figure 4D). Sunitinib was thus interrupted after 5 weeks and antibiotics were started, with progressive resolution of the infection, as confirmed by CT and PET three months later (Figure 4F). At that time, CT confirmed the tumor response to sunitinib with evidence of >30% shrinkage, thus consistent with a RECIST response (Figure 3C). The patient was not on treatment anymore, due to the complicated tumor response. A new progression was observed 5 months after treatment interruption (6 months from baseline), as shown by PET and CT in Figure 2C and Figure 3D. Thus sunitinib 37.5 mg/day was re-established, achieving a new CT response one month later (Figure 3E).

Following this experience another 67-years old consecutive patients, ECOG PS 1, affected by a *EWSR1*-*CHN* translocated progressive EMCS, metastatic to the lung and LN, pretreated with multiple lines of chemotherapy, started sunitinib in October 2011. After 3 months of treatment with sunitinib 37.5 mg/day a tumor disease stabilization was observed. At that point sunitinib was tentatively increased to 50 g/day, with a dimensional response by CT detectable after 3 months of therapy, 6 months from baseline.

Both patients are still on treatment and responsive, 11 and 8 months from baseline, respectively, without evidence of further major side effects.

## Discussion

We report on the tumor response to sunitinib in two consecutive patients with pretreated, progressive, metastatic extraskeletal myxoid chondrosarcoma, carrying the *EWSR1*-*CHN* translocation. In the first case the response was evident after only one month of treatment and marked by shrinkage of all lesions, on the face of a complete PET response. Decrease in tumor size was confirmed by further CT scans for 4 months after stopping treatment because of a therapy-related abscess. When disease progressed after 4 months from sunitinib discontinuation, response was restored by restarting treatment. At 9+ months of follow-up, tumor response was confirmed. In the second patient a dimensional response was evident at 6 months, after an initial tumor stabilization.

EMCS is a very rare STS, with low sensitivity to cytotoxic chemotherapy [15,20-24]. The only responses to chemotherapy were reported by McGrory in 2 of 6 metastatic EMCS patients responsive to a multi-agents chemotherapy [21], and in one patient treated with anthracycline plus ifosfamide described by Han [24]. No objective responses were observed in the MD Andersen series [23] of 10 patients receiving doxorubicin and dacarbazine-based regimens, nor in the series of 21 patients treated with different regimens, mostly anthracycline-based, reported by Memorial Sloan Kettering and Royal Marsden [20]. Finally, a response to interferon-α-2b was also described [27]. To our knowledge, this is the first report on the activity of an antiangiogenic agent in EMCS.

Sunitinib is a multi-targeted tyrosine-kinase (RTK) inhibitor and antiangiogenic drug approved for the treatment of gastrointestinal stromal tumor (GIST) and renal cancer [28,29]. Evidence of activity in selected STS subtypes other than GIST has been provided [30-34]. Responses are often non dimensional, with some exception as for alveolar soft part sarcoma (ASPS) [30]. Of interest, in these patients with ESMC we could observe a major dimensional response.

Unlike GIST, no specific genetic alterations associated with sensitivity to sunitinib have been identified in STS. As shown in ASPS [30], another STS bearing a translocation, the antitumor activity of sunitinib is unlikely to be directly linked to the fusion-protein. In the absence of selective targets and known mechanisms of action, sunitinib antiangiogenic activity as well as an effect on the autocrine-paracrine PDGFR/VEGFR activation-loop have been advocated as possible explanations for its antitumor activity [30]. Even in EMCS the fusion-protein is unlikely to be related to sunitinib sensitivity [12]. Unfortunately, due to absence of untreated frozen material, we could not assess the RTK activation profile.

The most common toxicities with sunitinib are hand-foot syndrome, rash, fatigue, hypertension, hypothyroidism and diarrhea. It is known that the sunitinib and other antiangiogenetic agents can interfere also with the normal vasculature formation and, possibly, with the T-cell mediated immunity [28,35,36]. This can lead to rare complications, such as abscess formation [37-39]. In our first patient, the abscess originated at a site where there was no evidence of disease and was probably related to the presence of the foreign material for the femoral fixation. For the differential diagnosis, CT is viewed as the most accurate exam. In our case the presence of clear clinical signs of infection, as fever, leukocytosis, calor and redness of the skin, were of much help to rule out an isolated disease progression. A conservative approach was enough to heal the abscess and a drainage could be avoided. However, CT findings may sometimes be insufficient for the diagnosis and a biopsy can be necessary to rule out a disease progression.

## Conclusion

A tumor response to sunitinib was seen in two consecutive patients with EMCS. In one case the response was complicated by infection and was restored when treatment could be restarted after a while. Differential diagnosis of complicated tumor response versus tumor progression was crucial to continue with therapy in the first responding patient. Further prospective studies are needed to confirm these results and to better understand the molecular basis for the activity of sunitinib in this disease.

## Consent

Written informed consent was obtained from the patients for publication of this Case Report and any accompanying legend. A copy of the written informed consents are available for review by the Editor-in-Chief of this journal.

## Competing interests

Stacchiotti S Pfizer srl: travel coverage for medical meetings, research funding. Casali PG Pfizer spa: advisory, research funding.

## Authors’ contributions

SS, PD, SP, AG and PGC contributed to the conception and design, to the analysis and interpretation of data, to manuscript drafting PD and TN carried out the molecular study CM carried out the radiological evaluation and contributed to the analysis and interpretation of data AR contributed case material, and contributed to the analysis and interpretation of data All the authors read and approved the final manuscript
